# Data on dengue incidence in South-eastern Brazil, 2014–2018

**DOI:** 10.1016/j.dib.2020.105266

**Published:** 2020-02-08

**Authors:** Expedito Luna, Gerusa Figueiredo, José Levi, Sérgio Campos, Alvina Felix, Nathalia Souza, Walter Figueiredo, Angela Costa, Maria Cardoso, Claudio Pannuti

**Affiliations:** a Faculdade de Medicina/ Instituto de Medicina Tropical, Universidade de São Paulo, São Paulo, Brazil; b Serviço Especial de Saúde de Araraquara, Universidade de São Paulo, Araraquara, Brazil; c Faculdade de Saúde Pública, Universidade de São Paulo, São Paulo, Brazil

**Keywords:** Dengue, Incidence, Cohort study, Seroprevalence, Brazil

## Abstract

Data from the routine surveillance systems have been extensively used to estimate the incidence of dengue. However, routine surveillance data frequently underestimate the diseases’ incidence. Underreporting of dengue cases is related to the varying spectrum of its clinical presentation, with a large proportion of mild and asymptomatic infections, to its unspecific signs and symptoms, to the limitations of access to health care, and to the performance of the surveillance system itself [1–3].

In order to obtain accurate figures on dengue incidence, a cohort of children and adolescents was set up and followed during four years. The incidence of reported cases was used as a reference for the sample size calculation, which was stratified by age groups. A two-stage procedure was used to select the participants: census tracts were randomly selected, and within each one, a pre-determined number of children of each age group was randomly selected.

The parents or legal guardians of the participating children and adolescents provided a written informed consent. In the first home visit, they responded to a questionnaire containing data on socio-demographic characteristics, housing, access to water, sewage, and garbage collection. Also, during the first visit a blood sample of the participating child/adolescent was collected for dengue baseline serology. Beginning in the week after the enrolment, the parent or legal guardian that was designated in the first visit received weekly phone calls for fever surveillance. If the child/adolescent had fever during the week, a nurse was dispatched to the family's home to collect more detailed data on the fever episode and collect a blood sample for dengue diagnosis (IgG, IgM, NS1 and PCR). If the dengue diagnosis was confirmed, a medical appointment was scheduled, and another blood sample for confirmatory tests was collected. It was also agreed that in every anniversary of their participation, they would receive another visit for a blood collection for dengue serology, regardless if they had a fever episode or a confirmed dengue diagnosis during the previous year.

This article contains the description of the cohort's dataset. It is associated with the article published in Acta Tropica, under the title “A cohort study to assess the incidence of dengue, Brazil, 2014–2018” [4]. The associated article focused on the seroprevalence and incidence of dengue, and explored some associations between both outcomes and some explanatory variables.

**Table:** Specifications Table

Subject	Medicine, Infectious diseases
Specific subject area	Infectious diseases epidemiology
Type of data	Table
How data were acquired	Interviews with parents or legal guardians of participating children and adolescents in home visits. Medical appointments. Laboratory tests results conducted in blood samples collected during home visits and medical appointments.
Data format	Raw Edited & Analyzed
Parameters for data collection	Households selected in the sampling procedure. Home visits: - Parent/Legal guardian signed a written informed consent. - Child/adolescent provided a written assent. - Provided a blood sample for baseline serologic test. Fever informed in weekly phone calls. Home visit during/immediately after fever episodes: provided blood sample for dengue diagnosis. Confirmed dengue cases: medical appointment, blood sample collected.
Description of data collection	Enrolment home visit: - Interview with parent/legal guardian. Questionnaire on socio-demographic variables, housing, sanitation. - Blood sample for dengue IgG antibody test. - Dengue IgG antibodies were tested, using an ELISA assay, (Dengue ELISA IgG, FOCUS Technologies, Cypress, CA, USA), according to the manufacturer instructions. Home visits for fever surveillance: - Interview with parent/legal guardian. Questionnaire on signs and symptoms accompanying the fever episode. - Blood sample for acute dengue diagnosis (IgM antibodies, NS1 antigen, dengue PCR). - Dengue IgM antibodies were tested, using a commercial ELISA assay (DengueVirus IgM Capture DxSelect^**TM**^**)**, according to the manufacturer instructions. - NS1 protein assay was performed by using a commercial kit (Platelia DENV-NS1 Ag, Bio-Rad, Marnes-la- Coquette, France), according to the manufacturer's instructions. - RNA was extracted from respectively 0.4 mL of plasma on the automated platform iPrep using the PureLink Virus Kit (Life Technologies, Brazil) or 0.5 mL in the NucliSENS Easy-Mag (Biomerieux, Brazil). - Extracted nucleic acids were submitted to a one-step dengue generic real-time polymerase chain reaction employing primers and probe previously described and TaqMan Fast Virus 1-Step Master Mix (ThermoFisher, Brazil). - Reactive samples were typed by submitting extracted RNA to four type-specific reactions, using the same conditions as above but replacing generic primers/probe by type-specific reagents [5]. Annual visits on the anniversary of participation: blood sample for IG antibodies, as described above.
Data source location	Instituto de Medicina Tropical/Faculdade de Medicina, Universidade de São Paulo (Tropical Medicine Institute, of the University of São Paulo's Medical School) São Paulo, State of São Paulo Brazil Samples stored at the Virology Laboratory of the Tropical Medicine Institute. Latitude and longitude:23.55 S; 46.67 W
Data accessibility	Repository name: Mendeley Data Data identification number: n32bwynzr4.1 (D.O.I. 10.17,632/n32bwynzr4.1) Direct URL to data: https://data.mendeley.com/datasets/n32bwynzr4/1
Related research article	LUNA, EJA et al. A cohort study to assess the incidence of dengue, Brazil, 2014–2018 Published in Acta Tropica 204 (2020) 105,313 D.O.I. 10.1016/j.actatropica.2019.105313

**Table:** 

**Value of the Data** • Dengue is one of the major emerging infectious diseases worldwide. Global warming has led to the spread of insect vectors to more northern and southern latitudes. The present dataset tracks the incidence of dengue fever at a site initially characterized as low endemic. • These data may be useful to the dengue research community, to policymakers in the public health field, and to the industry developing products related to dengue prevention and control. • Our dengue cohort has provided data on the disease prevalence, incidence, diagnosis, clinical presentation and explored some associations between both outcomes and some explanatory variables. • These data can be compared to similar studies, and can be aggregated with other similar datasets, increasing the sample size. • The outcome of a large outbreak of dengue was documented

## 1 Data description

Table 1 presents the description of the dataset, with the list of variables and categories/types of responses. The variables were grouped according to the moment when data were collected: at baseline interview, annual visits on the anniversary of participation, and during the fever episodes. At the baseline interview, the procedures were presented to parents or legal guardians, and an invitation was made for their son/daughter to participate. In the event of agreement on participation, the Informed Consent Form was read and signed. For older children and adolescents their signature of the Assent Form was also collected. The questionnaire contained data on the characteristics of the household, number of people living in the household, number of rooms, and sanitation. A blood sample was drawn from the participating child/adolescent for dengue serology. The family was asked to designate the member that would receive the weekly phone calls for fever surveillance. Beginning in the week after enrolment, this family member started receiving weekly phone calls for fever surveillance. If the participating child/adolescent had fever, a nurse would come to the family's home to collect a blood sample for dengue diagnosis, and to fill a questionnaire on dengue signs and symptoms. If the child/adolescent had a dengue diagnosis, a medical appointment was set. During that appointment, an additional blood sample was collected. Every anniversary of their participation they would receive a visit for a blood sample collection, regardless of having had a fever episode during the year.

**Table 1:** Description of the dataset, dengue incidence study in Brazil, 2014–2018.

Variables	Categories
Baseline interview	

study_id	numerical
Birth date	date (dd/mm/yyyy)
Census tract	Census Tract Code
Interview date	date (dd/mm/yyyy)
Age	numerical
Sex	Male/female
Enrolled in school	Yes/No
School year	1 Nursery/2 Preschool/3 Elementary School/4 High School/5 Undergraduate Studies/6 Graduated Studies/99 Not applicable
School level of head of household	1 Nursery/2 Preschool/3 Elementary School/4 High School/5 Undergraduate Studies/6 Graduated Studies/99 Not applicable
Type of household	House/Apartment/Other Type
Apartment floor	numerical
Number of people in household	numerical
Number of living rooms	numerical
Number of bedrooms	numerical
Number of kitchens	numerical
Number of bathrooms	numerical
Linked to water network	Yes/No
Water from well	Yes/No
Water from trucks	Yes/No
Water from river	Yes/No
Linked to sewage network	Yes/No
Garbage collection destination	Public Collecting System/Neighborhood Dumpster/Burned/Buried/Other

Laboratory results for baseline and annual serology	

Baseline Serology Date	date (dd/mm/yyyy)
Baseline Serology result	Positive/Negative/Inconclusive
Date of Annual serology - Year1	date (dd/mm/yyyy)
Result of Annual serology - Year1	Positive/Negative/Inconclusive
Date of Annual serology - Year2	date (dd/mm/yyyy)
Result of Annual serology - Year2	Positive/Negative/Inconclusive
Date of Annual serology - Year3	date (dd/mm/yyyy)
Result of Annual serology - Year3	Positive/Negative/Inconclusive

Laboratory results for each fever episode	

Fever Date Blood Draw	date (dd/mm/yyyy)
ELISA IgG Result	Positive/Negative/Inconclusive
ELISA IgM Result	Positive/Negative/Inconclusive
NS1 Result	Positive/Negative/Inconclusive
RT PCR Result	Positive/Negative
Genotype	DENV1/DENV2/DENV3/DENV4
Confirmed dengue diagnosis	1-Yes 0-No

Signs and symptoms for each fever episode	

Days of fever	numerical
Date of onset	date (dd/mm/yyyy)
Conjunctival injection	Yes/No
Headache	Yes/No
Abdominal pain	Yes/No
Myalgia	Yes/No
Arthralgia	Yes/No
Chills	Yes/No
Retro-orbital pain	Yes/No
Coryza	Yes/No
Cough	Yes/No
Sore throat	Yes/No
Diarrhea	Yes/No
Nausea	Yes/No
Vomiting	Yes/No
Intense vomiting	Yes/No
Somnolence	Yes/No
Restlessness	Yes/No
Bleeding	Yes/No
Rash	Yes/No
Itching	Yes/No
Loss of appetite	Yes/No
Axillary temperature	Numerical

The dataset contains also some new variables that were created from the original ones, to facilitate the analysis. They are:

a) Confirmed dengue case – 0/1 – if the participant had a fever episode in which acute dengue was diagnosed.
b) DENV 1–0/1 – for the ones who had an acute dengue diagnosis, if the genotype was dengue virus 1 (DENV1).
c) DENV 2–0/1 – for the ones who had an acute dengue diagnosis, if the genotype was dengue virus 2 (DENV2).
d) Asymptomatic dengue infection – 0/1 – if the participant had a dengue seroconversion detected in any of the annual serologic surveys, and did not have a fever episode in which the dengue diagnosis was confirmed.
e) Final date – necessary to calculate the follow up time.

Table 2 presents the cohort's planned sample size. The data on age-specific incidence of reported cases were used to estimate the sample size, stratified into four age groups (2–5 years of age, 5 to 9, 10 to 13, and 14 to 16).

**Table 2:** Planned sample size of the cohort study, stratified by age.

Age stratum	Sample size
2–4	1.463
5–9	1.018
10–13	525
14–16	405
Total	3.411

Three criteria were applied confirm dengue as the aetiology of the fever episodes. Table 3 presents the distribution of the confirmed cases according to them. The majority of cases were confirmed by the detection of dengue virus RNA by RT-PCR. A small proportion was confirmed by the detection of dengue virus NS1 antigen in serum samples, and just four cases were confirmed by seroconversion of IgM and IgG antibodies.

**Table 3:** Distribution of dengue incident cases according to diagnosis criteria.

Criterion	Number	%
RT – PCR	267	86.7
NS1	37	12.0
IgM and IgG seroconversion	4	1,3
Total	308	100.0

Table 4 presents the frequency of symptoms and signs among the 308 confirmed dengue cases. The more frequently reported symptoms were loss of appetite, myalgia, and abdominal pain. The frequency of warning signs, such as intense vomiting and fluid accumulation was low.

**Table 4:** Frequency of signs and symptoms among the confirmed dengue cases, Araraquara, 2014–2018.

Sign/Symptom	Number	%
Fever	308	100.0
Conjunctival injection	127	41.2
Headache	260	84.4
Abdominal pain	165	53.6
Myalgia	184	59.7
Arthralgia	146	47.4
Chills	125	40.6
Retro-orbital pain	128	41.6
Coryza	70	22.7
Cough	72	23.4
Sore throat	83	26.9
Diarrhea	59	29.2
Nausea	149	48.4
Vomiting	114	37.0
Intense vomiting	2	0.6
Somnolence	240	77.9
Restlessness	12	3.9
Bleeding	8	2.6
Rash	74	24.0
Itching	63	20.5
Loss of appetite	215	69.8
Petechiae	54	22.0^a^
Tourniquet test positive	2	0.8^a^
Hematocrit >45%	16	5.2
Leukocytes <3000	60	19.5
Platelets <150,000	38	12.3
Clinical fluid accumulation	4	1.6^a^
Fluid accumulation confirmed by ultrasound	3	1.2^a^
Total	308	100.0

a Of 245 subjects for whom the data is available.

## 2 Experimental design, materials, and methods

### 2.1 Design

In order to overcome the limitations of routine surveillance systems in estimating the burden of dengue [[1], [2], [3]] a cohort was set up to determine dengue incidence in children and adolescents from 2 to 16 years of age [4].

### 2.2 Site

It was conducted in Araraquara (Population 212,617 in 2012), a city located in the central region of the State of São Paulo (21°47′ S; 48°10’ W), a location classified as a mid level endemic location for dengue. The first autochtonous dengue case in the city was reported in 1995, and the largest outbreak prior to the period of observation occurred in 2011, when 2500 confirmed dengue cases were reported.

### 2.3 Period of follow up

Recruitment of participants was carried out from September 2014 to March 2015. The follow up begun in September 2014 and ended in December 2018.

### 2.4 Sample size

Dengue incidence in Brazil peaks in young adults [6] and is low among children. The age-specific incidence of dengue reported cases was used as a reference for the sample size calculation. The planned sample size can be seen in Table 2.

### 2.5 Sample selection procedure

A two-stage strategy was used to select the actual participants. In the first stage census tracts were randomly selected. In the selected census tracts the households with children and/or adolescents in the age range of interest were identified. Then, a pre-determined number of children/adolescents was randomly selected.

### 2.6 Ethical issues

The protocol was approved by the research ethics review board of the Hospital das Clínicas, of the University of São Paulo's Medical School, and is registered at the National Research Ethical Evaluation System, approval number CAAE25706913.6.1001.0065. The parents or legal guardians of the participating children and adolescents provided a signed Informed Consent. Older children and adolescents provided a written Informed assent.

### 2.7 Procedures

Selected households received a first home visit in which the cohort's aims and procedures were presented to the parents or legal guardians, and the invitation to the participation of their son/daughter was made. When they agreed to participate, informed consent and assent were obtained, and a questionnaire was filled, as previously described.

After enrolment, the designated family member received weekly phone calls asking if the child/adolescent has or had a fever during the week. A clinical thermometer was given to the participating family. From the second year on, they could opt for weekly text messages, instead of calls. When the participant reported a fever episode, a nurse would visit the family's home to collect a blood sample for acute dengue diagnosis, and fill a questionnaire on the signs and symptoms accompanying the fever. When the dengue diagnosis was confirmed, a medical appointment was scheduled. During that appointment another blood sample was collected. On every anniversary of their participation they received another nurse's visit for a blood collection for dengue serology.

### 2.8 Definitions

*Dengue suspect case*: any fever case, with axillary temperature above 37.5°.

*Symptomatic dengue confirmed case*: a suspect case with at least one of the following criteria fulfilled in acute and/or convalescent serum sample: 1: Detection of dengue RNA by real time polymerase chain reaction (RT-PCR). 2: Detection of NS1 protein. 3: Detection of IgM antibodies with IgG seroconversion. The number of cases confirmed by each criterion is presented in Table 3. The signs and symptoms presented by the confirmed dengue cases are presented in Table 4.

*Asymptomatic dengue infections* - Naïve subjects for dengue antibodies at baseline that presented seroconversion (at the one year serology sample) that did not report a febrile episode during the year were considered unapparent dengue infections. The same procedure was applied for those who seroconverted during the four-year follow up.

### 2.9 Outcomes

The main outcomes of the cohort follow-up were the dengue baseline seroprevalence, the annual seroprevalence, the cumulative incidence and incidence density of symptomatic confirmed dengue cases. Dengue baseline seroprevalence was calculated as the proportion of positive results in the recruitment sample. Yearly seroprevalence was calculated as the proportion of positive results in the yearly serologic surveys. Symptomatic confirmed dengue incidence was calculated as the number of symptomatic confirmed dengue cases divided by the population at risk: in the first year, the number of enrolled participants. For the following years, the number of subjects that participated in the yearly surveys was used as the denominator. Incidence density was calculated as the number of confirmed dengue cases in the numerator divided by the person-time units in the denominator. Asymptomatic dengue infections incidence was calculated as the number of asymptomatic dengue infections divided by the population at risk.

### 2.10 Laboratory methods

Dengue IgM and IgG antibodies were tested, using an ELISA assay (DengueVirus IgM Capture DxSelect^**TM**^ and Dengue ELISA IgG, FOCUS Technologies, Cypress, CA, USA), according to the manufacturer instructions.

Blood was drawn in Plasma Preparation Tubes (PPT, Beckton-Dickinson, Brazil) and centrifuged within 4 hours. RNA was extracted from respectively 0.4 mL of plasma on the automated platform iPrep using the PureLink Virus Kit (Life Technologies, Brazil) or 0.5 mL in the NucliSENS Easy-Mag (Biomerieux, Brazil). On both methods, total nucleic acids were eluted into 50uL of buffer.

Extracted nucleic acids were submitted to a one-step dengue generic real-time polymerase chain reaction employing primers and probe previously described [7] and TaqMan Fast Virus 1-Step Master Mix (ThermoFisher, Brazil). Forty cycles of 95 °C 10 seconds - 60 °C 40 seconds were performed in the thermocyclers models 7300 or 75-00 Fast (Applied Biosystems). Ct values below 37 were considered reactive for dengue RNA. Reactive samples were typed by submitting extracted RNA to four type-specific reactions using the same conditions as above but replacing generic primers/probe by type-specific reagents [5].

NS1 protein assay was performed by using a commercial kit (Platelia DENV-NS1 Ag, Bio-Rad, Marnes-la- Coquette, France), according to the manufacturer's instructions.

## Authors statement

**EJA Luna**: conceptualization, methodology, formal analysis, writing – original draft preparation, writing – review and editing. **GM Figueiredo**: conceptualization, methodology, writing – review and editing. **JE Levi**: methodology. **SRSLC Campos**: data curation, validation. **AC Felix**: investigation. **NS Souza**: investigation. **WM Figueiredo**: project administration, supervision. **AA Costa**: investigation, project administration, supervision. **MRA Cardoso:** conceptualization, methodology. **CS Pannuti:** conceptualization, funding acquisition, methodology, project administration, writing – review and editing.
